# Joseph Price

**Published:** 1911-07

**Authors:** 


### BUFFALO MEDICAL JOURNAL

**A Monthly Review of Medicine and Surgery**

*EDITOR*

WILLIAM WARREN POTTER, M. D.

All communications, whether of a literary or business nature, books for review and exchanges, should be addressed to the editor, 238 DELAWARE AVENUE, BUFFALO, N.Y.

VOL. LXVI. JULY, 1911. No. 12

### Joseph Price

IN the death of Joseph Price the world has lost one of its most brilliant abdominal operators and a man of force and character whose individuality and work will remain monuments to his memory as a gynecic surgeon.

Joseph Price had firmly fixed ideas regarding methods and manners of operating and whatever opinion any individual may have formed regarding his procedures there could be no question concerning his ability as a diagnostician in his chosen field of endeavor or his success as an operator. From the day when, as resident physician at the Philadelphia Dispensary, he laid the foundation of his fame by a large series of operations with uniformly successful results, until the day of his tragic death, Price occupied a position of enviable prominence. By his worth he demanded recognition while still a young man and he maintained his position to the time of his death on June 6, at his home in Philadelphia.

For two weeks Dr. Price had been ill and was confined to his bed most of the time. The day of his death he was informed that a girl patient whom he had previously cared for had been received at his hospital suffering with appendicitis. He rose from his sick bed and did an appendectomy on this patient. Within an hour after the completion of the operation he complained of pain and informed his assistants that he was suffering with acute appendicitis and that an immediate operation was imperative. He superintended the preparations for the work and in a short time was operated. A few hours later he died not having recovered from the shock of the operation.

Joseph Price was born in Rockingham County, Va., January 1, 1853. His early education was received in local schools and in New York state. He entered the medical department of the University of Pennsylvania being graduated with that famous class of 1877 which contained so many men who have achieved great prominence in medicine and surgery. He became resident physician of the Philadelphia Dispensary and was later placed in charge of the department of gynecology. It was during this time that he pointed for his prominence as a gynecologist and abdominal surgeon. He performed a great many operations in the alleys and tenements of Philadelphia under the most adverse circumstances and achieved brilliant successes. The long, almost unbelievable number of cases operated and the large number of recoveries reported brought to him a fame which has never dimmed. Later he was resident physician at the Preston Retreat.

Shortly after he entered upon private practice he established the Gynecean Hospital for the care of his patients and it became one of the best known institutions in this country. He was one of the founders of the American Association of Obstetricians and Gynecologists and its president in 1896.

In spite of the demands upon his time by his practice as a surgeon and consultant Dr. Price contributed much to the literature of appendicitis and gynecology and his writings are accepted as authority in these branches.

Although he visited Buffalo infrequently Dr. Price had many friends in this city and was the teacher of several Buffalo physicians who took special courses in gynecologic surgery at his private hospital in Philadelphia.

A widow and seven children survive him. A brother, also a well known gynecologist, Dr. Mordecai Price, died in 1904.

